# Supplementary material for: Spectral and non-spectral EEG measures in the prediction of working memory task performance and psychopathology
Source: bioRxiv. 2026 Mar 25:2026.03.25.714248. Preprint. [Version 1] doi: 10.64898/2026.03.25.714248 (PMC13042055; doi:10.64898/2026.03.25.714248)
Supplement: Supplement 1 [file NIHPP2026.03.25.714248v1-supplement-1.pdf]

bioRxiv preprint doi: https://doi.org/10.64898/2026.03.25.714248; this version posted March 26, 2026. The copyright holder for this preprint (which was not certified by peer review) is the author/funder, who has granted bioRxiv a license to display the preprint in perpetuity. It is made available under aCC-BY 4.0 International license.

Supplementary material

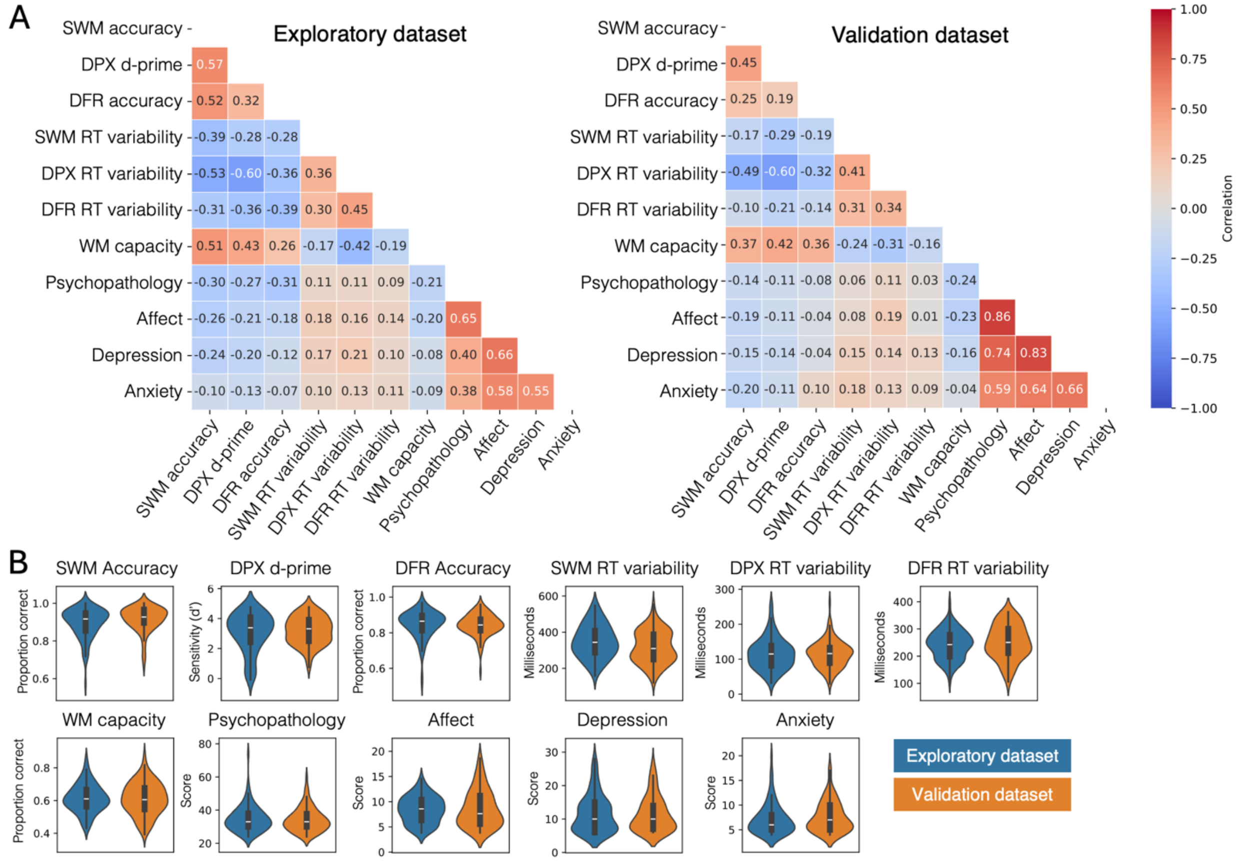

Supplemental Figure S1: Relationship among behavioral outcome measures and comparison across datasets.
(A) Heatmaps showing Pearson correlations between behavioral outcome measures within the exploratory (left) and validation (right) datasets. Each cell reflects the correlation between a pair of outcome variables. (B) Violin plots showing the distribution of each outcome measure in the exploratory (blue) and validation (orange) datasets. The width of each violin reflects the probability density of the data. Inner boxplots indicate the median and interquartile range. Distributions of all measures were comparable between the exploratory and validation datasets (all FDR-corrected $p > .05$).

bioRxiv preprint doi: https://doi.org/10.64898/2026.03.25.714248; this version posted March 26, 2026. The copyright holder for this preprint (which was not certified by peer review) is the author/funder, who has granted bioRxiv a license to display the preprint in perpetuity. It is made available under aCC-BY 4.0 International license.

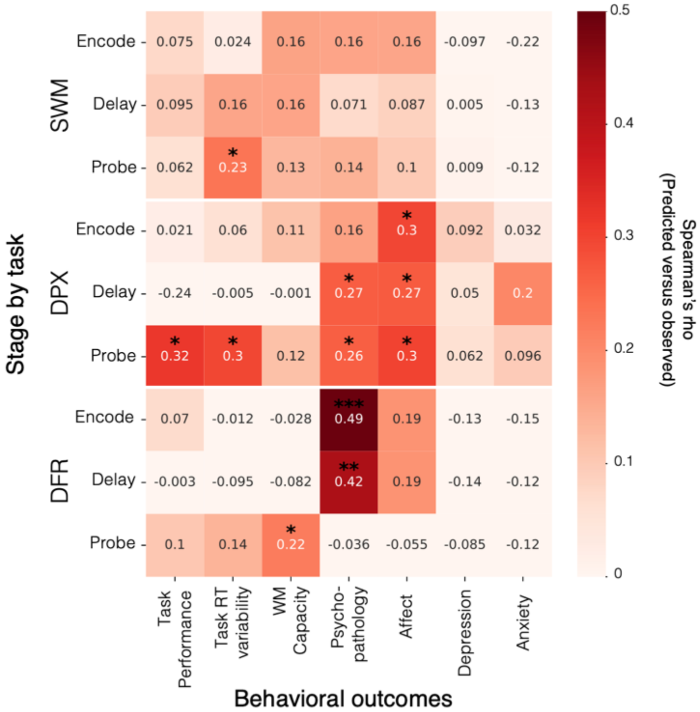

Supplemental Figure S2: Exploratory stage-specific model performance
Heatmap of the Spearman correlation between observed and predicted values for models trained with EEG features from individual stages of each task (y-axis) for different behavioral outcome variables (x-axis). Color reflects strength of correlation that is also denoted in the center of each cell. *p<0.05; **p<0.01; ***p<0.001.

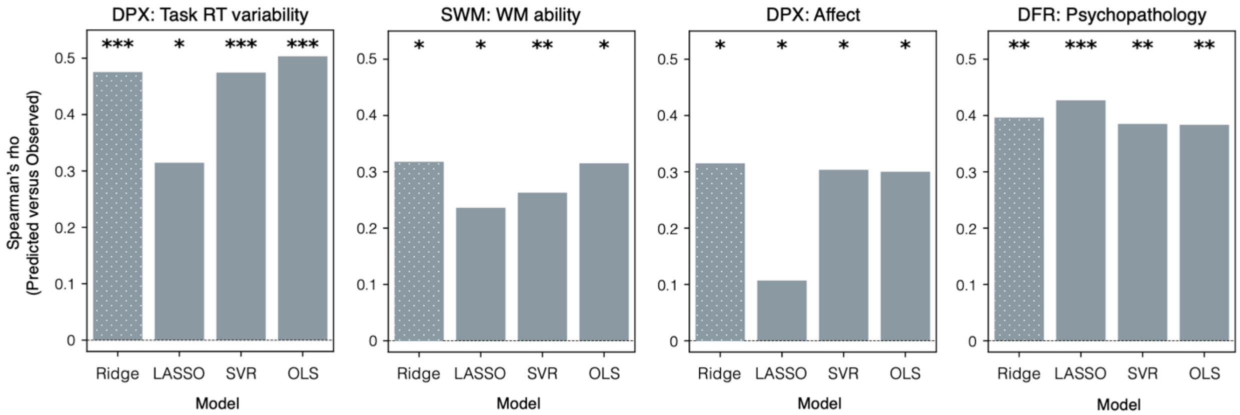

Supplemental Figure S3: Comparison of machine learning model performance
Performance for each main text model tested on held-out data using alternative regression approaches: ridge regression (dotted patten; model reported in main text), LASSO, support vector regression (SVR), and ordinary least squares (OLS). *p<0.05; **p<0.01; ***p<0.001.

bioRxiv preprint doi: https://doi.org/10.64898/2026.03.25.714248; this version posted March 26, 2026. The copyright holder for this preprint (which was not certified by peer review) is the author/funder, who has granted bioRxiv a license to display the preprint in perpetuity. It is made available under aCC-BY 4.0 International license.

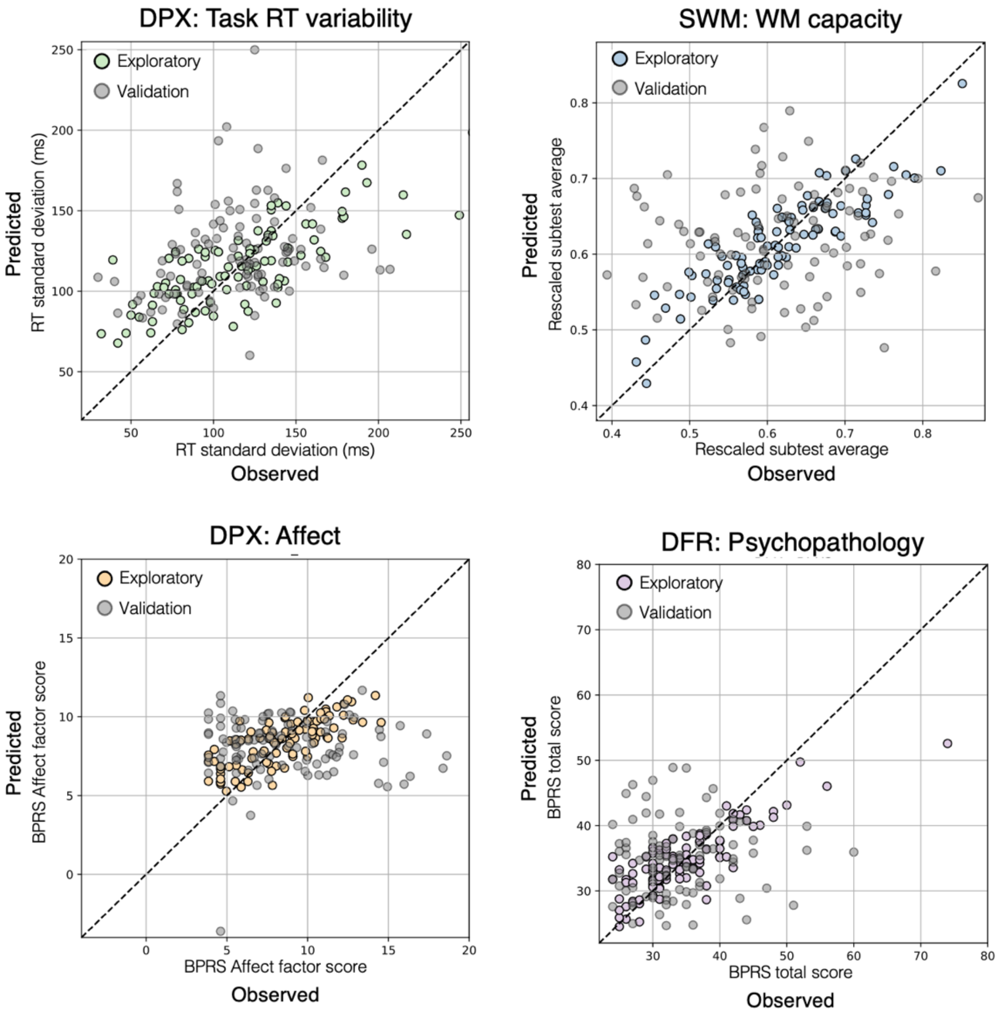

Supplemental Figure S4: Scatterplots of validated models.

Each plot shows observed versus predicted values for participants in the exploratory dataset used to train the model (colored circles) and participants in the validation dataset used to test the model (gray circles). Observed values are plotted on the x-axis and predicted values on the y-axis. Axis units correspond to the respective behavioral outcome. The diagonal dashed line indicates perfect correlation.
